# The Impact of HCV Infection Duration on HIV Disease Progression and Response to cART amongst HIV Seroconverters in the UK

**DOI:** 10.1371/journal.pone.0132772

**Published:** 2015-07-30

**Authors:** Jamie Inshaw, Clifford Leen, Martin Fisher, Richard Gilson, David Hawkins, Simon Collins, Julie Fox, Ken McLean, Sarah Fidler, Andrew Phillips, Sam Lattimore, Abdel Babiker, Kholoud Porter, UK HIV Seroconverters Cohort

**Affiliations:** 1 MRC Clinical Trials Unit at University College London, London, United Kingdom; 2 Western General Hospital, Edinburgh, United Kingdom; 3 Brighton and Sussex University NHS Trust, Brighton, United Kingdom; 4 Department of Infection and Population Health, University College London, London, United Kingdom; 5 Chelsea and Westminster Hospital, London, United Kingdom; 6 HIV i-Base, London, United Kingdom; 7 Guy’s and St. Thomas NHS Trust at Kings College, London, United Kingdom; 8 Charing Cross Hospital, London, United Kingdom; 9 Imperial College NHS Trust, London, United Kingdom; 10 Public Health England, London, United Kingdom; University of Montreal Hospital Research Center (CRCHUM), CANADA

## Abstract

**Introduction:**

The effect of HCV infection on HIV disease progression remains unclear; the effect of HCV infection duration on HIV disease progression is unknown.

**Methods:**

We used data from a cohort of HIV seroconverters to investigate the effect of HCV infection duration on time from HIV seroconversion to CD4 <350cells/mm^3^, AIDS or death, censoring at the earlier of cART initiation or last clinic visit, adjusting for confounders and splitting data into follow up periods from HIV seroconversion (<2, 2–4 and >4 years). We additionally compared CD4 cell decline following HCV infection to that of mono-infected individuals with similar HIV infection duration by fitting a random effects model. In a separate analysis, we used linear mixed models to we examine the effect of HCV infection and its duration on CD4 increase over 48 weeks following cART.

**Results:**

Of 1655 individuals, 97 (5.9%) were HCV co-infected. HCV<1 year was associated with a higher risk of endpoint in each follow-up period from HIV seroconversion (HR [95% CI] 2.58 [1.51, 4.41], p = 0.001; 3.80 [1.20, 12.03], p = 0.023; 2.03 [0.88, 4.71], p = 0.098 for <2, 2–4 and >4 years respectively), compared to mono-infected individuals. However, we found no evidence of an association for those with HCV>2 years (all p>0.89). Individuals experienced a somewhat greater decrease in CD4 count following HCV infection lasting 13 months, relative to individuals with HIV alone, (estimate = -3.33, 95% CI [-7.29, 0.63] cells/mm^3^ per month, p = 0.099). Of 1502 initiating cART, 106 (7.1%) were HCV co-infected, with no evidence of HCV duration at cART being associated with immunological response (p = 0.45).

**Conclusions:**

The impact of HCV co-infection on HIV disease progression appears to be restricted to the first year after HCV infection.

## Introduction

The impact of Hepatitis C Virus (HCV) on Human Immunodeficiency Virus (HIV) disease progression remains unclear. Due to the shared routes of transmission, 25–30% of HIV positive individuals are co-infected with HCV globally [1] and 9% in the UK [2].

Studies examining whether HCV affects HIV disease progression in the absence of combination antiretroviral therapy (cART) have generally been restricted to examining the effect on all-cause mortality, and reported no evidence of an effect [3–9]. However, given HIV-related causes of death are less common in co-infected individuals [9], investigating all-cause mortality may mask the effect of HCV on HIV-specific causes of death. Therefore it remains unclear whether co-infection affects HIV disease progression.

Given that CD4 cell count decreases in the presence of an opportunistic infection [10], HCV infection may be expected to affect CD4 cell decline in HIV positive individuals, given that HCV infection acts as an opportunistic infection in HIV positive individuals [11]. However, the immunological response to HCV infection may be longer-lasting, as HCV is a chronic disease. Understanding CD4 cell evolution in co-infected individuals is important given the increased incidence of HCV in HIV positive men who have sex with men (MSM) since 2005 [12, 13].

Furthermore, it is unclear how HCV affects immunological response to cART as, although the majority of studies, including a meta-analysis, report worse immunological response for co-infected individuals [4, 14, 15], some studies have found no such effect [5, 16–19]. The difference in these findings may be due to duration of HCV infection at cART initiation for co-infected individuals, as longer HCV infection duration may have a more profound effect on CD4 response. The relationship between the two viruses is important to understand as it may guide the optimal time of treatment initiation for co-infected individuals.

We aimed to examine the effect of HCV infection duration on HIV disease progression during ART-naïve follow-up, and on CD4 cell evolution following the initiation of cART. We used a national cohort of individuals with well-estimated dates of HIV seroconversion for whom we also estimated timing of HCV infection.

## Methods

The UK HIV Seroconverters Cohort is a national cohort of individuals whose time of HIV seroconversion has been reliably estimated. The cohort has been described in full elsewhere [20] but, briefly, the main inclusion criterion is that individuals have a negative and a positive HIV test at most 12 months apart. The cohort was set-up in 1994 and is followed up annually by collecting data from routine clinic visits.

Additional HCV data were obtained by matching patients from the UK HIV Seroconverters Cohort to data from the Sentinel Surveillance Study of Hepatitis and HIV testing in England, a study that collects information on hepatitis C testing carried out in 21 participating sentinel centres in the UK [21].

### Ethics Statement

The West Midlands – South Birmingham National Research Ethics Service specifically approved the study (04/G2707/155). Written informed consent was obtained from all participants enrolled after 2005. The ethics committee did not require written consent from participants already enrolled at that stage. No incentives were given for participation in the study.

### Statistical methods

#### Natural history

We examined whether HCV infection (Polymerase Chain Reaction (PCR) positive, or antibody positive, in the absence of PCR results) was associated with time to a composite endpoint of CD4<350 cells/mm^3^, AIDS defining condition, or death. In Cox proportional hazards models, we investigated the impact of HCV co-infection on time from HIV seroconversion to the composite endpoint, censoring at the earlier of cART initiation or date last seen before 6^th^ August 2014. HCV infection was fitted as a binary time-updated covariate in the model, allowing for clearing HCV. In multivariable analyses, we adjusted for age group (per 10 year increase), sex, diagnosis during acute HIV infection, risk group and decade of HIV seroconversion. We considered individuals to have entered the risk set once their HCV status was known. Therefore, individuals were excluded from the analysis if they had unknown HCV status throughout follow-up.

In order to examine the effect of HCV infection duration on time to the composite endpoint, we first fitted a Cox proportional hazards model including an interaction term between HCV duration and follow-up time to determine whether any effect of HCV duration varied over time from HIV seroconversion. As the interaction term was of borderline significance (p = 0.086), we split the data into follow-up periods (≤2, 2–4, >4 years from HIV seroconversion) to ensure HCV infected individuals were being compared to individuals with similar duration of HIV infection. We then fitted 3 Cox proportional hazards models. Duration of HCV infection was treated as a time-updated variable (≤1 year, 1–2 years, >2 years, and HCV uninfected).

In sensitivity analyses, we excluded CD4 measurements from the first 3 months after HIV seroconversion as CD4 cell count around that time are known to fluctuate widely [22]. Additionally, anti-HCV treatment was not recorded, so in order to allow for the possible short-term effect on CD4 cell count, we repeated the analysis insisting on a confirmed CD4 cell count <350 cells/mm^3^ as part of the composite endpoint, with an interval of >6 months between the two CD4 measurements. As individuals with faster decline may be more likely to initiate cART and, therefore, have censored follow-up, we repeated the analysis but included initiation of cART as a component of the composite endpoint. Finally, we repeated the analysis but excluded individuals who only had antibody positive HCV results and no PCR data.

We also investigated ART-naïve dynamics of CD4 cell count evolution by comparing CD4 cell decline of co-infected individuals following estimated HCV infection date to that of mono-infected individuals with similar duration of HIV. This was done by fitting a random effects mixed model with a knot, comparing the CD4 count following HCV infection to CD4 counts of HIV-monoinfected individuals with a CD4 measurement taken at a similar (<30 days) time point after HIV seroconversion. We adjusted for HCV status and the interaction between HCV status and time, as well as duration of HIV at baseline, age group (per 10 year increase), sex, diagnosis during acute HIV infection, risk group and decade of HIV seroconversion. We established the most appropriate time point to place the knot by choosing the model based on Akaike Information Criterion (AIC). For HCV co-infected individuals to be included in this analysis, they were required to have evidence of HCV infection (HCV negative followed by a positive HCV test) and at least 1 CD4 cell measurement after HCV infection and before cART initiation.

#### Response to cART

We investigated the impact of HCV infection at cART initiation on immunological recovery by fitting random effects mixed models examining CD4 count from cART initiation over 48 weeks of follow-up. In multivariable analyses, we adjusted for duration of HIV at cART initiation, HIV test interval, age group at cART initiation (per 10 year increase), sex, decade of cART initiation and risk group, as well as baseline CD4 count. A baseline reading was defined as any reading (or the mean if >1 reading) 3 months before to 2 weeks after cART initiation. The impact of duration of HCV at cART initiation on immunological response to cART was analysed by fitting the same model but with duration of HCV infection as a continuous variable instead of HCV infection as a binary variable. We included any individual with known HCV status with at least one baseline measurement and two follow-up CD4 measurements within 48 weeks from cART initiation. In a sensitivity analysis, we repeated the analysis but restricted to virally suppressed individuals, in order to compare individuals with and without HCV who are likely to have been adherent.

As information on HCV status was not always recorded, in all analyses we estimated HCV seroconversion dates based on the assumption that individuals received annual HCV tests from 2003 onwards, when annual testing for HIV positive individuals was recommended in the UK [23]. Table 1 summarises how this assumption was implemented for estimating HCV seroconversion dates. Additionally, we assumed that individuals with previous evidence of HCV co-infection who subsequently had two consecutive HCV negative PCR tests to have cleared HCV infection. In sensitivity analyses, we repeated all analyses without assumptions about annual HCV testing for individuals with missing HCV status. We instead regarded each individual’s HCV status as unknown until their first recorded HCV test, then assumed their HCV status changed on the day of the HCV test. In all analyses, we defined cART as any ART regimen started after 1^st^ January 2000; before 1^st^ January 2000, cART was defined as a 3 drug ART regimen containing drugs from 2 different classes, or 3 nucleoside reverse transcriptase inhibitors (NRTIs), provided Tenofovir or Abacavir were taken as part of that regimen.

**Table 1:** Assumptions around HCV infection date for HCV co-infected individuals in the UKR Register of HIV seroconverters.

HIV seroconversion Date*	First HCV positive Test	Assumed date of HCV infection
Before 01 January 2003	Before HIV seroconversion	HIV seroconversion date
	Later than or on the day of HIV Seroconversion and before 01 January 2003	Midpoint of last negative and first positive HCV test dates. If no negative test, then date of first positive test.
	Later than or on the day of HIV Seroconversion and after 01 January 2003	Midpoint of last negative and first positive HCV test dates, unless tests >1 year apart – in which case the midpoint of last assumed negative test date (annually from 01 January 2003 onwards) and first positive test.
Later than or on 01 January 2003	Before HIV seroconversion	Midpoint of last negative and first positive HCV test dates. If no negative test, then date of first positive test.
	Later than or at the same time as HIV Seroconversion	Midpoint of last negative and first positive HCV test dates, unless tests are >1 year apart – in which case the midpoint of last assumed negative test (annually 1 year after seroconversion date onwards) and first positive test.

*We assumed that routine testing for HIV positive individuals was taking place from 2003 according to BHIVA guideline

## Results

### Natural history

Of 1655 individuals eligible for the study, 1558 were HCV negative throughout follow-up and 97 (5.9%) had evidence of HCV infection at any time during follow-up; 42 with PCR positive tests, the remainder with antibody positive tests. Median (IQR) follow-up time until censoring was 1.16 (0.33, 3.27) years. HCV uninfected individuals were more likely to be male (94.8% vs. 80.4%), less likely to be people who inject drugs (PWID) (0.3% vs. 28.9%) and more likely to have seroconverted in later years (median [IQR] year of seroconversion 2006 [2002, 2010] vs. 2000 [1994, 2006]) compared to HCV co-infected individuals (Table 2). The composite endpoint was reached by 875 (56.2%) individuals who were HCV negative throughout follow-up (14 deaths, 21 AIDS defining illnesses, 840 CD4<350 cells/mm^3^) and 68 (70.1%) individuals who were HCV infected at any time up until the event/censoring date (1 death, 2 AIDS defining illness, 65 CD4<350 cells/mm^3^). Follow-up for 574 individuals was censored due to cART initiation (550 HCV negative vs. 24 HCV positive).

**Table 2:** Baseline characteristics of HIV seroconverters by HCV infection status.

	HCV negative N = 1558	HCV positive* N = 97	Total N = 1655
Male	1477 (94.8%)	78 (80.4%)	1555 (94.0%)
Risk group: MSM** ^†^	1406 (90.2%)	60 (61.9%)	1466 (88.6%)
MSW^††^	124 (8.0%)	7 (7.2%)	131 (7.9%)
PWID^†††^	4 (0.3%)	28 (28.9%)	32 (1.9%)
Unknown/other	24 (1.5%)	2 (2.1%)	26 (1.6%)
Ethnicity: White	1377 (88.4%)	77 (79.4%)	1454 (87.9%)
Black African	36 (2.3%)	0 (0.0%)	36 (2.2%)
Unknown/other	145 (9.3%)	20 (20.6%)	165 (10.0%)
Diagnosis during acute HIV infection***	91 (5.8%)	6 (6.2%)	97 (5.9%)
Year of HIV seroconversion: median (IQR)	2006 (2002, 2010)	2000 (1994, 2006)	2006 (2001, 2010)
Age at HIV seroconversion: median (IQR)	32.4 (26.9, 40.4)	31.5 (25.5, 37.9)	32.4 (26.9, 40.2)

*Evidence of HCV co-infection at any time up to time of the event/censoring date

**Includes 5 individuals who are also PWID

*** HIV positive test and negative test within 30 days of each another

^†^ Sex between men

^††^Sex between men and women

^†††^People who inject drugs

Individuals with HCV infection were more likely to reach the composite endpoint (HR = 1.57, 95% CI: [1.14, 2.16], p = 0.006). Becoming HIV-positive after 2010 was associated with a higher risk of endpoint (HR = 2.02, 95% CI: [1.15, 3.54], p = 0.014), while PWID had lower risk of endpoint (HR = 0.50, 95% CI: [0.30, 0.84], p = 0.010).

Table 3 shows results from analyses examining HCV duration by follow-up period. In each period, individuals with HCV infection <1 year had a higher risk of reaching the composite endpoint than individuals with HIV infection alone (HR [95% CI] 2.58 [1.51, 4.41], p = 0.001; 3.80 [1.20, 12.03], p = 0.023; 2.03 [0.88, 4.71], p = 0.098 for <2, 2–4 and >4 years respectively). Individuals with 1–2 years HCV infection also had a higher risk of reaching the endpoint, but only for those with 2–4 years of HIV infection (HR [95% CI] 0.59 [0.23, 1.51], p = 0.271; 3.56 [1.91, 6.62], p<0.001; 1.43 [0.34, 5.90], p = 0.624 for <2, 2–4 and >4 years respectively). There was no evidence that individuals with HCV for >2 years were at greater risk of reaching the endpoint than individuals with HIV mono-infection (HR [95% CI] 1.00 [0.31, 3.24], p = 0.994; 0.95 [0.45, 1.99], p = 0.888 for 2–4 and >4 years respectively).

**Table 3:** Effect of HCV co-infection on time from HIV seroconversion to CD4<350 cells/mm^3^, AIDS or death by HCV infection status and HCV infection duration: UK Register of HIV seroconverters.

Follow-up time (years)	HCV infection status/duration (years)			
	HCV uninfected	<1	1–2	>2 years
<2: Number of individuals (Number of events)	1448 (471)	34 (16)	18 (6)	19 (0)
Unadjusted hazard ratio [95% CI]	1	2.91 [1.77, 4.79]	0.80 [0.36, 1.79]	**
Adjusted* hazard ratio [95% CI]	1	2.58 [1.51, 4.41]	0.59 [0.23, 1.51]	**
2–4: Number of individuals (Number of events)	507 (196)	4 (3)	13 (11)	20 (6)
Unadjusted hazard ratio [95% CI]	1	3.53, [1.13, 11.06]	3.48 [1.89, 6.40]	0.69 [0.31, 1.56]
Adjusted* hazard ratio [95% CI]	1	3.80 [1.20, 12.03]	3.56 [1.91, 6.62]	1.00 [0.31, 3.24]
>4: Number of individuals (Number of events)	285 (208)	11 (6)	3 (2)	20 (18)
Unadjusted hazard ratio [95% CI]	1	2.06 [0.90, 4.70]	1.18 [0.29, 4.76]	0.62 [0.37, 1.01]
Adjusted* hazard ratio [95% CI]	1	2.03 [0.88, 4.71]	1.43 [0.34, 5.90]	0.95 [0.45, 1.99]

*Adjusted for sex, age at cART (per 10 years), diagnosis during acute HIV infection, decade of HIV seroconversion and risk group.

**Not possible to estimate the hazard ratio because the estimation algorithm did not result in convergence towards an estimate.

The results from all 5 sensitivity analyses investigating the impact of HCV infection duration on HIV disease progression remained qualitatively unchanged (data not shown).

**Fig 1:**
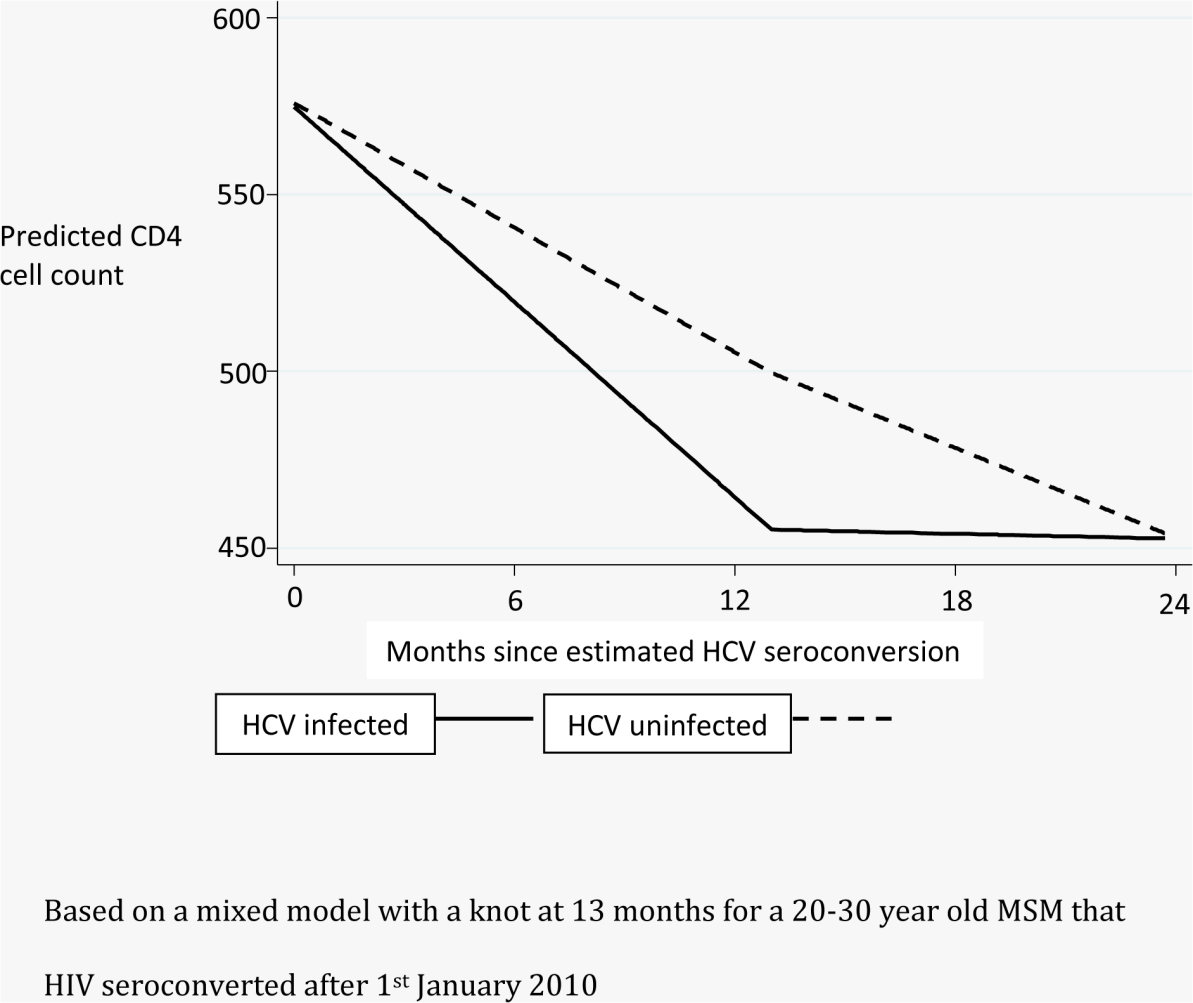
Predicted declines in CD4 counts comparing HCV infected individuals to those with similar HIV duration without HCV infection.

Investigating CD4 cell decline following HCV infection, 63 HCV infected individuals and 1720 HCV uninfected individuals were included in the analysis. The model with the lowest AIC was one with a knot placed at 13 months after HCV infection. There was borderline evidence to suggest that in the first 13 months after HCV infection, individuals with HCV experienced greater CD4 decline per month than individuals without HCV infection (estimate = -3.33, 95% CI [-7.29, 0.63] cells/mm^3^ per month, p = 0.099). This is equivalent to a fall over 13 months of 43.29 cells/mm^3^ more for an individual with HCV infection compared to an individual with HIV mono-infection. After 13 months, there was evidence of CD4 recovery in individuals with HCV co-infection and their CD4 count increased per month relative to individuals without HCV (estimate = 3.99, 95% CI [1.48, 6.50] cells/mm^3^ per month, p = 0.002). Fig 1 shows predicted CD4 cell evolution over 2 years after HCV infection for a 20–30 year old MSM that HIV seroconverted after 1^st^ January 2010.

### Response to cART

Of 1502 individuals eligible for analysis, 106 (7.1%) were HCV positive at cART initiation. HCV negative individuals were more likely to be male (94.7% vs. 77.4%), to seroconvert in later years (median [IQR] year of HIV seroconversion 2004 [1999, 2009] vs. 1994 [1987, 2001]), to have a shorter duration of HIV at cART initiation (median years [IQR] 2.35 [0.76, 5.44] vs 7.33 [2.78, 13.47]), to have higher baseline CD4 cell counts (median [IQR] 323.1 [242.4, 443.0] vs. 258.7 [165.5, 370.0] cells/mm^3^), and less likely to be PWID (0.6% vs. 48.1%) compared to co-infected individuals (Table 4).

**Table 4:** Baseline characteristics of HIV seroconverters initiating cART by HCV status.

	HCV Negative N = 1396	HCV Positive* N = 106	Total N = 1502
Male	1322 (94.7%)	82 (77.4%)	1404 (93.5%)
Risk Group: MSM^†^	1253 (89.8%)	49 (46.2%)	1302 (86.7%)
MSW^††^	119 (8.5%)	5 (4.7%)	124 (8.3%)
PWID^†††^	8 (0.6%)	51 (48.1%)	59 (3.9%)
Unknown/other	16 (1.1%)	1 (0.9%)	17 (1.1%)
Ethnicity: White	1226 (87.8%)	72 (67.9%)	1298 (86.4%)
Black African	47 (3.4%)	0 (0.0%)	47 (3.1%)
Unknown/other	123 (8.8%)	34 (32.1%)	157 (10.5%)
Years between HIV seroconversion and cART initiation: Median (IQR)	2.35 (0.76, 5.44)	7.33 (2.78, 13.47)	2.51 (0.84, 5.82)
Years HIV test interval: median (IQR)	0.58 (0.29, 0.97)	0.84 (0.38, 1.27)	0.60 (0.29, 1.00)
Year of seroconversion: Median (IQR)	2004 (1999, 2009)	1994 (1987, 2001)	2004 (1999, 2008)
Age at first cART Median (IQR)	37.11 (30.76, 44.23)	37.48 (33.55, 42.33)	37.13 (30.90, 44.04)
Baseline CD4: Median (IQR)	323.1 (242.4, 443.0)	258.7 (165.5, 370.0)	320.0 (238.5, 438.3)

*Evidence of HCV co-infection at cART initiation

^†^ Sex between men

^††^Sex between men and women

^†††^People who inject drugs

HCV co-infected individuals experienced lower CD4 increase following cART initiation (estimate = -0.90, 95% CI [-1.81, 0.01] cells/mm^3^ per week, p = 0.053) equivalent to 43.28 [95% CI 87.10, -0.54] cells/mm^3^ lower over 48 weeks. Individuals starting cART in later years were more likely to have a better CD4 cell response than individuals who started cART before 2000 (estimate [95% CI] = 38.1 [16.7, 59.4], p<0.001; 46.5 [23.0, 70.0], p<0.001 for 2000–2010 and >2010 compared to pre-2000, respectively). As expected, higher baseline CD4 count was associated with higher CD4 cell gains (estimate = 0.94 per cell, 95% CI [0.90, 0.98] cells/mm^3^, p<0.001) and longer HIV infection duration at cART initiation was associated with lower CD4 cell counts following cART initiation (estimate = -0.08 per week, 95% CI [-0.11, -0.05] cells/mm^3^, p<0.001). The effect of HCV co-infection was stronger when restricting the analysis to individuals with viral suppression (estimate [95% CI] = -1.52 [-2.75, -0.28] cells/mm^3^ per week, p = 0.016).

We found no overall evidence to suggest that HCV infection duration at cART initiation was associated with CD4 cell gains over 48 weeks, even after restricting to individuals who were virally suppressed (p = 0.45 and p = 0.27 respectively).

Results from all sensitivity analyses where assumptions about annual HCV testing were dropped remained qualitatively unchanged, with the exception of the CD4 cell count following HCV infection, which gave similar estimates but without a significant association, likely due to a lack of statistical power (data not shown).

## Discussion

This is the first study to investigate the effect of HCV infection duration on HIV disease progression and response to cART. We found that, in the absence of cART, there is evidence of shorter time to our composite endpoint for HCV-infected, compared to HCV uninfected individuals. However, this seems to be restricted to the first year or so following HCV infection. This is supported by our finding of greater CD4 loss for HCV co-infected individuals in the first thirteen months after HCV infection, which is followed by compensatory CD4 cell recovery.

These findings can, therefore, be regarded as consistent with studies which have reported no impact of HCV co-infection on all-cause mortality [3–9]. They may also explain why some studies have found no overall effect of HCV infection on CD4 cell evolution [3, 7], as any such effect is likely only in the first year following HCV infection. One study reported no overall difference in immunological progression between HCV co-infected and HIV mono-infected individuals, but significantly poorer progression for co-infected individuals with CD4 >600 cells/mm^3^ [6]. Of interest, the HCV co-infected population in that study were largely PWID, who are likely to have contracted HCV infection at the same time as HIV and would, therefore, have had short HCV infection duration. Our finding of more rapid CD4 decline only in the first year of HCV infection would tend to lend support to this finding.

We found some evidence of different initial decline in CD4 cell count while ART-naïve following HCV infection. This may be due to the suggested higher levels of T-cell activation upon contracting HCV [24] and, therefore, an increased initial replication rate for both HCV and HIV.

On initiation of cART, we also found that HCV co-infected individuals have lower immunological gains than HIV mono-infected individuals. Certainly there is evidence that HCV may replicate in the same cells as HIV-1 [25], which may account for these observed lower CD4 cell gains. Our finding is supported by a number of studies [4, 9, 14], including a meta-analysis [15], although one study suggested there is no immunological impact of HCV infection following cART initiation [5]. The reasons for discrepancies in findings are unclear, but it has been suggested that duration of HCV may have been responsible for differences in findings [5]. However, we found no evidence to suggest that CD4 cell count rise following cART initiation was associated with HCV infection duration. It is likely, therefore, that this finding was due to other factors such as late ART initiation or different ART regimens.

There are a number of implications for clinical practice as a result of these findings. First, for patients presenting with recently-acquired HCV infection, a sustained fall in CD4 cell count is expected before recovering in the absence of cART. There is, therefore, no specific reason to preferentially initiate immediate cART, as in line with current guidelines [26]. This may be an important finding at a time when HCV incidence amongst HIV positive individuals is known to have increased [12, 13]. Secondly, individuals with HCV co-infection will have slower CD4 cell count recovery following cART initiation, regardless of duration of HCV infection duration. There is no immediate need, therefore, to start rather than delay cART initiation based on concerns of optimal CD4 cell recovery.

There are a number of limitations to our study, the most important being that we have limited/no information on HCV treatment and it may be argued that the drop in CD4 count observed in the first 13 months following HCV infection merely reflects the effect on CD4 cell count of anti-HCV treatment [27]. However, anti-HCV treatment is, at most, of 24-week duration [28] and, although we could not account for this in analysis, we performed a sensitivity analysis requiring a confirmed CD4 cell count <350 cells/mm^3^ >6 months after the initial reading as part of the composite endpoint. Furthermore, the sustained decrease in CD4 count of 13 months is longer than the 24-week duration of anti-HCV treatment. Finally anti-HCV treatment uptake is known to have been low during the study period [29–30], at 17% in the UK [31]. A further limitation is that the HCV infection dates were estimated using a number of assumptions based on annual HCV testing in UK clinics. These assumptions were removed in sensitivity analyses and findings remained qualitatively unchanged.

In conclusion, we found no evidence to suggest that HCV co-infection influences HIV disease progression beyond the initial time of co-infection with HCV. A drop in CD4 cell count at that stage is expected, which is sustained for about a year. HIV-HCV co-infected individuals however, experience poorer CD4 gains upon initiation of cART, regardless of duration of HCV at cART initiation.
